# Comparative analysis of Cdc48-dependent proteolysis at the ER, mitochondria and chloroplasts

**DOI:** 10.1038/s41467-026-74728-z

**Published:** 2026-07-02

**Authors:** Anne Sophie Lau, Sreedhar Nellaepalli, R. Paul Jarvis

**Affiliations:** Section of Molecular Plant Biology, Department of Biology, University of Oxford, South Parks Road, Oxford, UK

**Keywords:** Proteasome, Ubiquitylation, Endoplasmic reticulum, Mitochondria, Chloroplasts

## Abstract

The ubiquitin-proteasome system (UPS) is the preeminent proteolytic system in eukaryotes. While soluble nucleocytosolic proteins are readily accessed by the UPS, organelle-localised proteins present major, membrane-related accessibility challenges. Cells overcome this problem by employing the conserved AAA+ ATPase Cdc48 to extract organellar proteins to the cytosol, thereby enabling proteasomal degradation. Major Cdc48-dependent proteolytic systems exist at the endoplasmic reticulum, mitochondria and chloroplasts, and are uniquely adapted to deliver protein homeostasis within the respective organelles. We provide a focused comparison of these systems, analysing similarities and differences between them. Better understanding of underlying principles has important implications spanning human health and agriculture.

## Abstract (web summary)

The ubiquitin–proteasome system degrades proteins but faces accessibility barriers at organelles. Here the authors provide comparative insights across endoplasmic reticulum, mitochondria and chloroplasts, revealing shared mechanisms and organelle-specific adaptations shaping proteostasis.

## Introduction

**Fig. 1:**
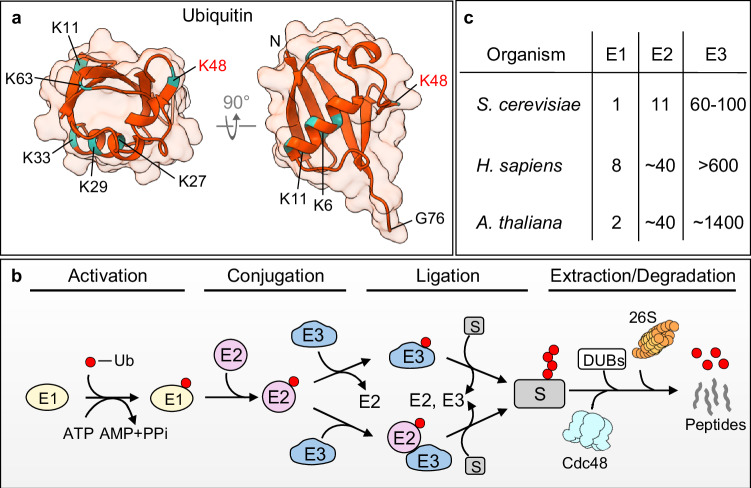
Ubiquitin and the ubiquitination cascade. **a** Two orientations of ubiquitin (PDB: 1UBQ) showing the distribution of lysine residues (K6, K11, K27, K29, K33, K48 and K63). K48 (highlighted in red) is the key site for the formation of K48-linked polyubiquitin chains, the canonical signal targeting proteins for degradation by the ubiquitin-proteasome system. During ubiquitination, the C-terminal glycine (G76) forms thioester bonds with ubiquitination enzymes, ultimately leading to covalent attachment to substrate proteins. **b** Ubiquitin (Ub) is first activated in an ATP-dependent manner by a ubiquitin-activating enzyme (E1). The activated ubiquitin is then transferred to a ubiquitin-conjugating enzyme (E2), before a ubiquitin ligase (E3) mediates the final transfer of ubiquitin to the substrate protein (S). The E3 ligases are primarily responsible for substrate specificity, often forming transient or stable complexes with the substrate and E2. Other factors, including deubiquitinases (DUBs), Cdc48, and the 26S proteasome (26S) (see Boxes 1 and 2), act downstream to deliver degradation of ubiquitinated substrates and recycle ubiquitin. **c** The complexity of the ubiquitination system varies considerably across species, as indicated by the approximate numbers of E1, E2 and E3 enzymes identified in *Saccharomyces cerevisiae* (yeast), *Homo sapiens* (human), and *Arabidopsis thaliana* (plant).

Protein homeostasis (proteostasis) is an essential aspect of cell function in living organisms, serving to fine-tune and balance protein synthesis, folding and degradation. In eukaryotes, the latter is orchestrated principally by the ubiquitin-proteasome system (UPS), a nucleocytosolic pathway that is highly conserved from yeast to photosynthetic organisms (including plants) and mammals^1–3^. In this system, proteins destined for degradation are marked (covalently tagged) with a small, 76-amino-acid polypeptide called ubiquitin (Fig. 1a).

**Fig. 2:**
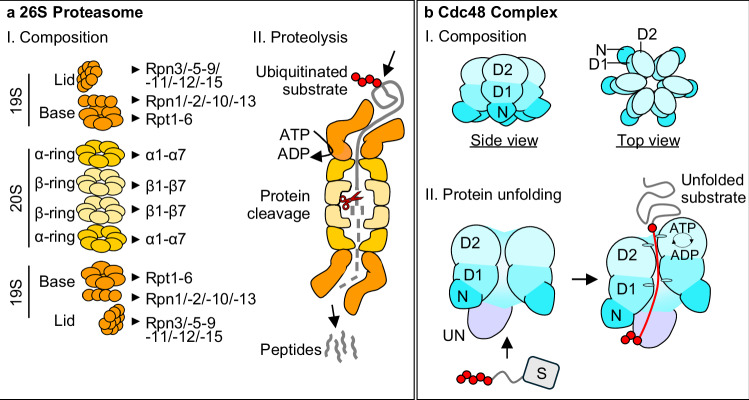
Overview of the 26S proteasome and the Cdc48 chaperone. **a** Structural arrangement of the 26S proteasome. (I) The proteasome is composed of numerous subunits, as indicated, which are organised into two distinct particles. The 20S core particle (CP) has a barrel-like structure and is capped, at one or both ends, by one or two 19S regulatory particles (RPs). (II) The RPs play crucial roles in the delivery of ubiquitinated substrate proteins into the interior of the CP for proteolysis. Further details are provided in Box 1. **b** Structural arrangement of the homohexameric Cdc48 complex. (I) Each Cdc48 monomer is composed of an N-terminal domain (N) and two ATPase domains (D1, D2), as well as a C-terminal tail not shown here. Side- and top-view schematic diagrams show the arrangement of these domains in the hexamer. (II) The Cdc48 complex cooperates with the Ufd1-Npl4 (UN) adaptor proteins, which facilitate recognition of ubiquitinated substrates (ubiquitin moieties are shown in red; the thick red and grey lines represent a partially unfolded ubiquitinated substrate polypeptide). The Cdc48 complex undergoes ATPase cycle-dependent conformational changes that enable a “spiral staircase” arrangement of axial loops to translocate the substrate through the central pore of the hexamer, thereby mediating extraction, unfolding and delivery to the cytosolic proteasome. In this mechanism, subunits of Cdc48 hydrolyse ATP one after another in a sequential process that produces directional movement of the unfolded polypeptide substrate.

The attachment of ubiquitin is called ubiquitination (or ubiquitylation) and is facilitated by a cascade of enzymes. First, ubiquitin is activated by a ubiquitin-activating enzyme (E1) in an ATP-dependent reaction, forming a ubiquitin-E1 thioester bond. The activated ubiquitin is then transferred to a ubiquitin-conjugating enzyme (E2), forming another thioester. Finally, the ubiquitin is transferred to the target protein (or substrate) with a ubiquitin ligase (E3) providing substrate specificity. Ubiquitin transfer from the E2 to the substrate can occur either directly or via the E3 as an intermediate (Fig. 1b)^4,5^. Ubiquitin attachment occurs primarily at lysine residues, resulting in substrate monoubiquitination or, more commonly, polyubiquitination. In the latter, each ubiquitin unit is typically attached to one of several lysines in the preceding ubiquitin unit, resulting in the formation of unbranched or branched polyubiquitin chains^5,6^. Because substrate specificity is conferred by the E3 (so that only target proteins are marked for degradation), there are over 600 E3 ligases in humans and over 1400 E3 ligases in plants (*Arabidopsis thaliana*), whereas only a few E1s and E2s exist in each case (Fig. 1c)^7–10^. Subsequently, the ubiquitin-labelled proteins enter and are degraded by a large, multi-subunit proteolytic machine called the 26S proteasome (Box 1, Fig. 2a)^11,12^, with the ubiquitin molecules being released for subsequent re-use through the action of deubiquitinases (DUBs)^13^.

While soluble nucleocytosolic proteins are readily available to the 26S proteasome, multimeric protein complexes, integral membrane proteins, and organelle-localised proteins require an additional energy-dependent extraction or retrotranslocation step prior to their degradation. This physical extraction process is orchestrated by the protein-unfoldase activity of a AAA+ ATPase called Cdc48 (Box 2, Fig. 2b)^14,15^. The active Cdc48 motor complex is a homohexameric ring, with each Cdc48 monomer possessing N-terminal, double ATPase, and C-terminal tail domains. The complex works in concert with several cofactors that contribute to substrate recognition and unfolding. The Cdc48-catalysed extraction step is essential to expose inaccessible proteins and make them available for degradation by the 26S proteasome^16–18^.

**Fig. 3:**
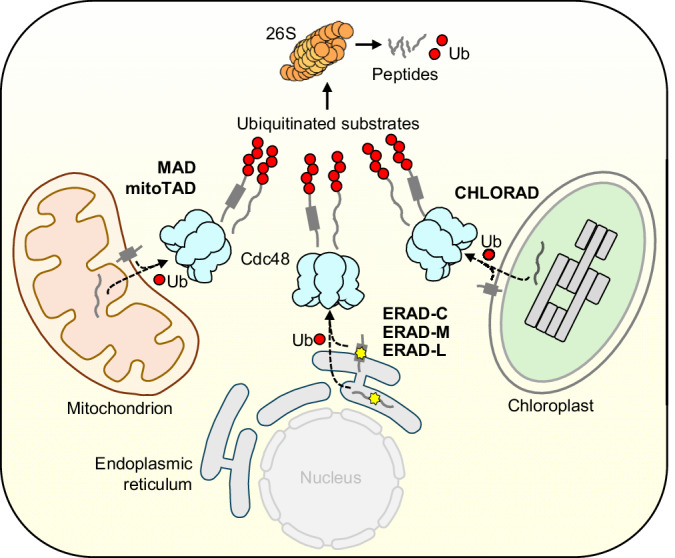
Overview of Cdc48-dependent proteolytic pathways targeting major organelles in eukaryotic cells. The AAA+ ATPase Cdc48 (known as p97/VCP in mammals) plays a key role in the ubiquitin-proteasome system by facilitating the extraction of polyubiquitinated substrates from organelles to enable their subsequent degradation by the 26S proteasome (26S) in the cytosol. At the endoplasmic reticulum (ER), Cdc48 acts on ER proteins with misfolded domains in the lumen (ER-associated protein degradation-L; ERAD-L), membrane (ERAD-M), and cytosol (ERAD-C). At mitochondria, Cdc48 acts on proteins integrated in the outer mitochondrial membrane (mitochondria-associated protein degradation; MAD) as well as precursor proteins arrested within the protein import apparatus (mitochondrial protein translocation-associated degradation; mitoTAD). At chloroplasts in plant cells, Cdc48 acts on components of the translocon of the outer chloroplast membrane (TOC) to regulate protein import (chloroplast-associated protein degradation; CHLORAD). In each case, the relevant Cdc48-dependent pathway acts to prevent the accumulation of misfolded proteins or to remove proteins that are surplus to requirements for regulatory reasons. Emerging evidence suggests that MAD and CHLORAD may also regulate proteins in internal compartments of the respective organelle, highlighting the expanding repertoire of Cdc48 in maintaining proteostasis in eukaryotic cells.

The endoplasmic reticulum (ER), mitochondria, and chloroplasts (in photosynthetic eukaryotes) have acquired distinct ubiquitin-mediated proteolytic systems that are specifically adapted to the differing demands of each organelle (Fig. 3). The most well-characterised system, ER-associated protein degradation (ERAD), was first identified in the 1990s and has since been studied extensively^19–23^. More recently, mitochondria-associated protein degradation (MAD), mitochondrial protein translocation-associated degradation (mitoTAD), and chloroplast-associated protein degradation (CHLORAD) have been discovered as key processes for maintaining proteostasis within the mitochondria and chloroplasts, respectively (Table 1)^24–27^. Although not considered in detail here, analogous systems have also been identified in lipid droplets (LDAD) and at the inner nuclear membrane (INMAD)^28–31^. Each of these UPS-dependent pathways is focused around a key role for Cdc48 in substrate protein retrotranslocation, although is specialised for the unique challenges posed by the relevant organelle whilst also contributing to overall cellular proteostasis.

**Table 1:** Comparative overview of the ERAD, MAD, mitoTAD and CHLORAD systems

	ER	Mitochondrion	Chloroplast
**Boundary**	Single membrane	Double membrane	Double membrane
**Main substrates**	Proteins with a misfolded domain in the ER lumen (L), membrane (M), cytosol (C)*	OMM proteins (MAD), arrested precursor proteins (mitoTAD)	OEM proteins (TOC components)
**Ubiquitination cascade**	E1: Uba1 (L, M, C) E2: Ubc7 activated by Cue1, Ubc6, also Ubc1 (all L, M, C) E3: Hrd1 (L, M), Doa10 (C)	E1: Unknown^†^ E2: Unknown^†^ E3: Rsp5 (MAD, mitoTAD), Mdm30, Ubr1, San1 (all MAD), Mfb1 (F-box protein; MAD)	E1: Unknown^†^ E2: Unknown^†^ E3: SP1, SPL2
**Retrotranslocon**	Hrd1 (L, M), Der1 (L), Dfm1 (M), Doa10? (C)	Tom40	SP2
**Cofactors**	Ufd1, Npl4, Ubx2 (all L, M, C), Hrd3 (L, M), Usa1 (L, M?), Yos9, Mnl1-Pdi1 (all L)	Ufd1, Npl4, Ubx2 (all MAD, mitoTAD), Doa1 (MAD)	PUX10
**DUBs**	Otu1 (L)	Ubp2, Ubp12 (both MAD), Ubp16 (mitoTAD)	Unknown^†^
**Ubiquitin-chain linkage**	K48 (L, M, C)	Unknown^†^	Unknown^†^

*Note that these abbreviations (L, M, C) are used to indicate the relevant ERAD pathway(s) in each of the following cells.

^†^The indicated components or features have not yet been determined through experimental analysis.

The different pathways are summarised and compared according to the various points discussed in detail in the text.

In this article, we consider these ER, mitochondrial and chloroplast systems together in the context of a broader discussion on the role of the UPS in organellar proteolysis. We compare and contrast the different systems to reveal common principles and unique adaptations that enable them to function in specific subcellular contexts. Exploring similarities and differences between the pathways has clear potential to improve our understanding of the UPS more broadly, which will be essential to address important issues in human disease and agriculture.

Box 1 The 26S proteasome The 26S proteasome, a 2.5 MDa multiprotein complex, is the nucleocytosolic ATP-dependent protease acting at the end of the UPS pathway. It is highly conserved in eukaryotes and consists of two functionally-distinct stable subcomplexes: the 20S core particle (CP) and the 19S regulatory particle (RP), with the latter associating peripherally with the core in one or two copies. The 20S CP is composed of four stacked heteroheptameric rings (two outer α-rings comprising subunits α1-α7, and two inner β-rings comprising subunits β1-β7) that form a barrel-shaped structure^170–172^. Upon assembly, the two β-rings at the centre form a catalytic chamber harbouring six catalytic sites that are responsible for cleaving peptide bonds in substrate proteins. Access to this proteolytic chamber is tightly controlled by apical gates formed by the α-rings located at either side of the β-rings. In the absence of 19S particles, the α-ring gates remain closed, preventing unregulated entry of proteins into the chamber^172–175^.

The 19S RP is required for substrate recognition, deubiquitination, unfolding, and translocation into the 20S core. It consists of two distinct subcomplexes: a base subcomplex proximal to the CP α-ring, and a horseshoe-shaped lid subcomplex. The base subcomplex contains a ring of six regulatory particle AAA+ ATPase subunits (Rpt1-Rpt6) and four regulatory particle non-ATPase subunits (Rpn1, Rpn2, Rpn10, Rpn13). The ATPase subunits form a hexameric ring and use the energy from ATP hydrolysis to unfold and translocate substrate proteins through an axial pore into the 20S core; whereas the non-ATPase subunits function in ubiquitin recognition and binding. The outermost lid complex is composed of nine non-ATP subunits (Rpn3, Rpn5-Rpn9, Rpn11, Rpn12, Rpn15) and helps to connect the 19S particle to the core, with the DUB Rpn11 being involved in ubiquitin recycling^172,176–179^.

Proteolysis begins with the high-affinity binding of a ubiquitinated substrate to the ubiquitin-receptor Rpn subunits of the 19S RP, leading to conformational changes in the adjacent α-ring of the CP and the opening of the gated axial pore. The Rpt ring then engages an unstructured region of the substrate protein and employs ATP hydrolysis to mechanically unfold the substrate. As the substrate is unfolded, it is threaded through the open α-ring pore into the proteolytic chamber of the 20S CP. During this process, Rpn11 cleaves the ubiquitin chain from the substrate, ensuring that only the substrate but not ubiquitin is internalised. Inside the chamber, the substrate encounters the proteolytically active β-subunits that cleave the substrate into short peptides, which are then released into the cytosol and further degraded to amino acids by various peptidases^172,178,180^. Although our understanding of protein degradation by the 26S proteasome has matured considerably in recent years through the application of structural approaches and biochemical analysis, much remains to be explored concerning how the proteasome interacts with other pathways, such as the ERAD, MAD, mitoTAD and CHLORAD systems.

Box 2 The hexameric AAA+ ATPase Cdc48 The Cdc48 (p97/VCP in mammals) protein is a highly-conserved and essential molecular chaperone, and a member of the AAA+ (ATPase associated with diverse cellular activities) family of ATPases^181^ – like the Rpt subunits of the 19S RP. The Cdc48 protein plays important roles in numerous cellular processes, especially those related to protein homeostasis. It leverages its ATPase-powered unfoldase activity to extract, disassemble and unfold ubiquitinated substrates from macromolecular complexes and membrane compartments (such as the organelles that are targeted by the ERAD, MAD, mitoTAD and CHLORAD pathways) to enable their degradation by the 26S proteasome in the cytosol^78,79,122,123,146^.

The operational structure of Cdc48 is a homohexameric ring complex. Each subunit possesses an N-terminal domain (NTD) and two conserved AAA+ ATPase domains (D1 and D2) stacked in tandem, and in the hexamer, the latter form two ATPase rings with a central axial pore that is critical for substrate translocation and unfolding. The ATPase domains contain Walker A and Walker B motifs that mediate ATP binding and hydrolysis^77,182–184^. Hydrolysis of ATP occurs in a sequential, coordinated manner around the hexamer, with the D1 ring primarily involved in substrate engagement and initial translocation steps and the D2 ring providing the main mechanical force driving substrate unfolding. Coordinated movement of the ATPase domains is thought to mediate substrate threading through the central pore via a “spiral staircase” mechanism^77,185^. The spiral staircase is formed of multiple pore loops of the ATPase domains, which contain conserved aromatic residues. These grip and pull on substrate polypeptides in a stepwise fashion, driven by ATP-dependent conformational cycling, to translocate the substrate through the central pore. The pore loops also act as a molecular ratchet to prevent substrate backsliding, thereby ensuring unidirectional translocation^77,185,186^. At the periphery of the complex are the NTDs, which are flexible and mediate interactions with numerous cofactors and substrates^182^. The NTDs adopt multiple conformations to accommodate different cofactors, often undergoing rotations or positional shifts relative to the ATPase rings during the ATPase cycle, thus coordinating substrate recruitment with processing. The Cdc48 protein additionally possesses a conserved C-terminal extension (or tail) of around 50 amino acids, which contributes to the regulation of ATPase activity and serves as a binding site for a subset of cofactors^182^.

The broad versatility of Cdc48 is largely attributable to its interaction with a diverse set of cofactors. An extraordinary number of cofactors have been identified to date and are known to confer specificity by directing Cdc48 to different substrates, subcellular locations, or biological processes^14,15^. These cofactors can be categorised into three functional groups: substrate recruitment factors, which bind to ubiquitinated substrates and facilitate their delivery to Cdc48; substrate processing factors, which assist in remodelling and unfolding of the substrates; and regulatory cofactors, which modulate the ATPase activity of Cdc48^82^. Most cofactors interact with the NTD of Cdc48, although a minority of them have been shown to interact with the C-terminal tail.

### Endoplasmic reticulum protein degradation pathways

The ER is a multifunctional organelle and the main entry gate for secretory protein biogenesis. Protein homeostasis in the ER is essential for the coordination of many functions, and this relies on ERAD, which selectively targets misfolded or unassembled proteins for degradation by the 26S proteasome^23^. Deficiencies in ERAD cause the accumulation of misfolded proteins, leading to cellular damage. Thus, it is not surprising that ERAD has been implicated in several diseases including neurodegenerative disorders such as Parkinson’s disease, where impaired clearance of defective proteins leads to disruption of neuronal function^32–34^.

**Fig. 4:**
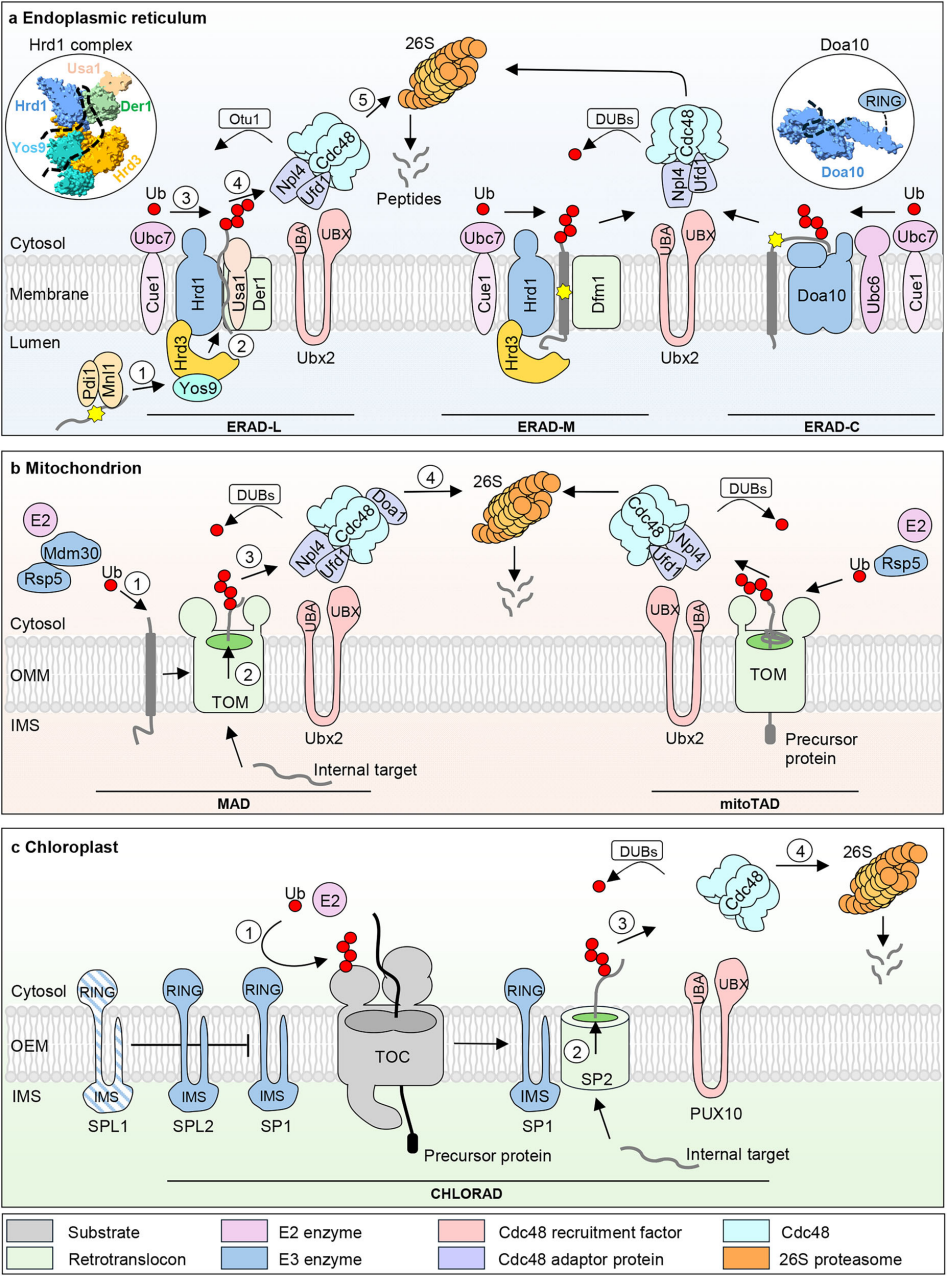
Detailed comparison of Cdc48-dependent proteolytic systems targeting the ER, mitochondria and chloroplasts. Each panel summarises knowledge of the relevant Cdc48-dependent proteolytic systems, including deubiquitination by deubiquitinases (DUBs), and proteasomal degradation. **a** ERAD is categorised into three branches based on the location of the misfolded substrate domain (represented by the yellow star). The ERAD-L branch (left side) targets substrates with a misfolded luminal domain via the Mnl1-Pdi1 complex (1) and the Hrd3-Yos9 recognition complex (2). Hrd1 and Der1 form the retrotranslocation channel, with Hrd1 (E3) and Ubc7 (E2) mediating ubiquitination (3). Then, Cdc48-Ufd1-Npl4 (Cdc48-UN), recruited by Ubx2, extracts the substrate from the membrane (4), enabling proteasomal degradation (5). The inset shows a cryo-EM structure of the Hrd1 complex for ERAD-L, highlighting the presumed translocation path with the substrate polypeptide (PDB: 6VJZ, 6VK3). In the ERAD-M branch (middle), proteins with a misfolded membrane domain are ubiquitinated by Hrd1 (E3) and Ubc7 (E2), and exported via Dfm1 with assistance from the Cdc48-UN complex prior to proteasomal degradation. In the ERAD-C branch (right side), substrates with a misfolded cytosolic domain are targeted by the Doa10 machinery, composed of Doa10 (E3), Ubc6 and Ubc7/Cue1 (E2s), and extracted via Cdc48-UN prior to proteasomal degradation. The inset shows a cryo-EM structure of the Doa10 protein, highlighting the lateral tunnel with the substrate polypeptide (PDB: 8TQM). **b** In MAD (left side), proteins in the outer mitochondrial membrane (OMM) are ubiquitinated by E3 ligases (including Rsp5 and Mdm30), working in concert with yet unknown E2s (1). Substrates are retrotranslocated through the Tom40 protein-import channel (2), energised by Cdc48-UN, which is recruited to the OMM by Ubx2 (3). Doa1 may contribute to Cdc48 recruitment or substrate recognition. Finally, ubiquitinated substrates are degraded by the proteasome (4). Internally localised proteins may also be targeted by MAD. In mitoTAD (right side), precursor proteins arrested in Tom40 are ubiquitinated by Rsp5 (E3), and extracted with the assistance of Cdc48-UN, which is again recruited by Ubx2, prior to their proteasomal degradation. **c** In CHLORAD, components of the TOC machinery are targeted by SP1 and SPL2 (E3s), working with yet unknown E2s (1). Ubiquitinated substrates are retrotranslocated via the channel protein SP2 (2), with the assistance of Cdc48, which is recruited to the chloroplast by PUX10 (3), prior to their proteasomal degradation (4). Activity of SP1 is subject to negative regulation by SPL1. Internally localised proteins may also be targeted by CHLORAD.

Two key findings initially uncovered the link between ER protein degradation and the UPS: (1) genetic studies in yeast revealed the participation of an E2 enzyme in the turnover of a mutant form of the ER translocon component Sec61p; and (2) inhibitor studies in mammalian cells revealed that the degradation of mutant, ER-mislocalized cystic fibrosis transmembrane conductance regulator (∆F508 CFTR) is UPS-dependent^19–22,35^. Subsequent discoveries revealed that ERAD is conserved across eukaryotes and can be classified into branches based on the location of the substrate or its misfolded domain^23^. In yeast, the most studied pathways are ERAD-L (lumen), ERAD-M (membrane), and ERAD-C (cytosol) (Fig. 4a), all of which are discussed individually below^36^. Studies have also provided insights into more specialised ERAD pathways, including ribosome-associated ERAD (ERAD-RA) and translocon-associated ERAD (ERAD-T)^37–40^.

The ERAD system has been extensively studied in yeast, and accordingly, the components discussed in the following sections are referred to using the nomenclature of that system.

### Degradation of proteins with a misfolded domain in the ER lumen (ERAD-L)

Targets of ERAD are ubiquitinated at the organelle surface by different E3 ligases acting in concert with several E2 ubiquitin-conjugating enzymes such as Ubc7. The latter is recruited to the ER and activated by Cdc48/Ufd1-interacting element 1 (Cue1), leading to the formation of lysine 48 (K48)-linked polyubiquitin chains as the signal for target degradation^41–44^. In ERAD-L, substrates are primarily engaged by a RING-type E3 ubiquitin ligase, hydroxymethyl glutaryl-coenzyme A reductase degradation protein 1 (Hrd1), which additionally possesses a multi-spanning membrane domain that acts as a translocase (Fig. 4a)^45–48^.

Hrd1 forms a complex in which at least two specialised substrate-recognition factors act to ensure selective degradation of only terminally misfolded proteins: yeast OS-9 homologue (Yos9), a lectin protein which specifically recognises mannose-trimmed N-glycans on ER glycoproteins (a hallmark of prolonged ER residence and failed folding)^49,50^; and Hrd3 (SEL1L in mammals), which acts as a scaffold in the Hrd1 complex, supports complex stability, and binds unstructured or misfolded regions of target polypeptides^51,52^. The N-glycan signal is created by the sustained action of glycosidases (in particular, stepwise mannosidase cleavage), ultimately exposing α1,6-linked mannose adjacent to the glycan core, a prerequisite for identification by Yos9 (OS-9 in mammals)^53,54^. This, together with the unfolded nature of the polypeptide, targets substrates to the Hrd1 complex; and so there is a dual signal (glycan-based and polypeptide conformational) for substrate selection in ERAD.

Recent studies revealed that initiation of ERAD involves a luminal bifunctional complex consisting of mannosidase like protein 1 (Mnl1; also called Htm1) and protein disulfide isomerase (Pdi1)^55^. The Mnl1-Pdi1 complex trims substrate glycan chains, as already described, and may reduce disulfide bonds to generate an unfolded polypeptide that can be transported across the ER membrane (Fig. 4a)^55^. In addition, several luminal chaperones, including the heat shock protein 70 (Hsp70)-type factor Kar2, act to maintain substrate solubility and facilitate selection for ERAD^56,57^. Retrotranslocation of substrates is mediated by Hrd1 in cooperation with a rhomboid pseudoprotease, degradation in ER 1 (Der1), with U1-Snp1-associating 1 (Usa1) performing a scaffolding function in this retrotranslocon complex^36,58–60^.

Structural analysis using cryo-EM revealed the architecture and mechanistic details of the monomeric Hrd1 complex that functions in ERAD-L^48^. In this study, the entire Hrd1 complex was assembled as a model by analysing two subcomplexes (Hrd1-Usa1-Der1-Hrd3 and Hrd1-Hrd3-Yos9), on the basis of overlapping protein composition. In the complex, Hrd3 and Yos9 mutually create a luminal binding site that detects aberrant folding and glycan modification of substrate proteins. Following recognition, substrates are delivered to the Hrd1 retrotranslocon. In this part of the complex, Hrd1 forms a “half-channel” with a cytosol-facing cavity, and Der1 forms another “half-channel” with a lumen-facing cavity, the two being juxtaposed in a thinned, distorted membrane region^48^. The lateral gates of Hrd1 and Der1 face each other, allowing substrates to pass from the lumen to the cytosol^48^. This retrotranslocation process is initiated by insertion of a substrate polypeptide loop into the ER membrane, with one segment of the loop contacting Hrd1 and another contacting Der1 (Fig. 4a inset)^48^.

### Degradation of proteins with a misfolded domain in the ER membrane (ERAD-M)

In ERAD-M, integral membrane proteins with a defect in a membrane-embedded region are targeted for degradation. Core components of the ERAD-L machinery are also involved in this pathway, including Hrd1, Hrd3 and likely also Usa1, but excluding the luminal recognition components and Der1^36,45,54,58,61,62^. Der1 may be replaced by a Der1 homologue called Der1-like family member (Dfm1), which functions in substrate retrotranslocation and Cdc48 recruitment (Fig. 4a)^63–65^. Far less is understood about substrate recognition in ERAD-M. It has been suggested that chaperones like Hsp70 and heat shock protein 40 (Hsp40) may help with maintaining the solubility of the substrates^66,67^.

Another membrane-focused phenomenon involves the clearance and degradation of proteins that obstruct the ER translocon (Sec61) and is termed ERAD-T. Proteins that cannot be fully translocated and become stalled in Sec61 are recognised through the exposure of unstructured regions. These substrates are then also targeted by Hrd1, but the relevant pathway is less well defined^37,68^. This is functionally analogous to the mitoTAD process in mitochondria (see later).

### Degradation of proteins with a misfolded domain at the cytosolic side of the ER (ERAD-C)

In ERAD-C, proteins possessing a defect in a cytosolic domain are targeted for degradation. This branch is defined by a different E3 ligase, degradation of alpha-2 10 (Doa10). The Doa10 protein was initially identified for its role in degrading a soluble transcription factor (Matα2), as well as ER proteins, revealing its ability to degrade soluble and membrane-anchored substrates^69^. Accordingly, it is localised in the inner nuclear membrane and the ER. Like Hrd1, Doa10 is a large multi-spanning membrane protein with a cytosol-facing RING domain, and it has been suggested to act in substrate retrotranslocation^70^.

Structural analysis by cryo-EM revealed an unusual architecture of Doa10. In this, the transmembrane domain forms a C-shaped (or horseshoe-like) structure with a large central cavity occupied by membrane lipids, while the conserved middle domain forms a lateral tunnel open to the cytosol^71^. Although the resolution of the cytosolic RING domain was low, it is placed over the tip of the horseshoe structure and opposite the lateral tunnel. This arrangement (lateral opening, central cavity, RING) may provide an optimal docking platform for selective substrate binding following degron entry into the lateral tunnel, ensuring that only substrates can access the central cavity for ubiquitination by the RING domain (Fig. 4a inset). It is suggested that the entry of a degron peptide (e.g., an exposed hydrophobic lesion) of the substrate into the lateral opening is crucial for polyubiquitination^71^.

Doa10 works in concert with the E2 enzymes Ubc6 (which is membrane-integrated) and Ubc7 (which is cytosolic and functionally dependent on Cue1, as already described for ERAD-L), forming K48-linked polyubiquitin chains that signal degradation^69,70,72^. Substrate recognition in ERAD-C is not well understood, though it also involves binding of misfolded substrate domains by cytosolic Hsp70/Hsp40 chaperones, helping to maintain substrates in a soluble, degradation-competent state^73,74^. Recent findings suggest that the Doa10 lateral tunnel forms a membrane-embedded entry path that directly engages exposed hydrophobic segments of substrates, enabling selective recognition within the lipid bilayer^71^.

### Convergence of the different ERAD pathways at Cdc48

After ubiquitination, the various ERAD branches converge at Cdc48, which mediates the energy-demanding step of substrate retrotranslocation prior to degradation in the proteasome. The Cdc48 protein works in a complex together with its cofactors, ubiquitin fusion degradation 1 (Ufd1) and nuclear protein localisation 4 (Npl4) (this complex is known as Cdc48-UN). The Ufd1 and Npl4 cofactors support substrate recognition and unfolding by Cdc48, and are essential for substrate processing prior to degradation. The Cdc48-UN complex is recruited to the ER by the UBX domain-containing protein 2 (Ubx2), which exposes its ubiquitin-associated (UBA) and a ubiquitin regulatory X (UBX) domains towards the cytosol^17,75–81^. The UBX domain provides a docking site for cytosolic Cdc48, while the UBA domain promotes interactions with ubiquitinated substrate proteins. In the final stages, DUB enzymes trim the ubiquitin chain, and the processed substrate is transferred to the proteasome for degradation. For ERAD-L at least, it has been shown that substrates are processed by ovarian tumour 1 (Otu1) and one or more other DUBs^17,82,83^.

### ERAD in plants

**Fig. 5:**
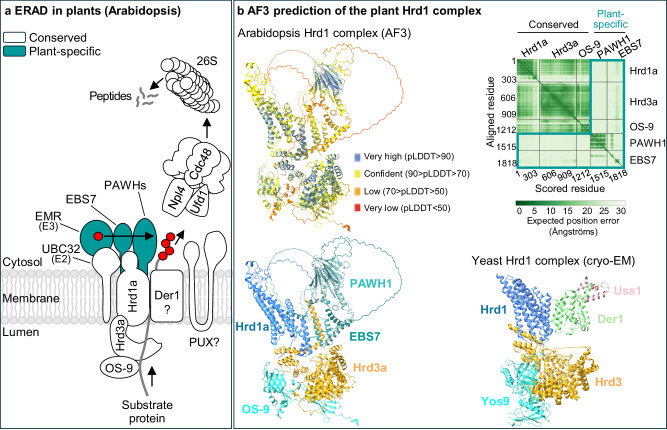
Composition and structural analysis of the ERAD machinery in plants. **a** Schematic diagram of the plant ERAD machinery, based on data from Arabidopsis. Individual components are colour-coded to identify those that are conserved and those that are plant-specific. **b** AlphaFold 3 (AF3) prediction of Arabidopsis Hrd1 complex. Composition of the complex is based on the discussed interaction data, and so Der1 is ommitted. The structural model is coloured according to pLDDT confidence scores (top left) and the identity of the different polypeptides (bottom left). The corresponding predicted alignment error (PAE) plot is also shown (top right). Comparison of the plant model with the cryo-EM structure of the yeast complex (PDB: 6VJZ, 6VK3; bottom right) reveals both conserved and plant-specific features. The conserved features are predicted with high confidence scores.

While ERAD pathways and their underlying mechanisms are well studied in yeast and mammalian systems, our understanding of ERAD in plants is relatively limited^84,85^. Although the core apparatus of ERAD is well conserved, plants notably possess an expanded repertoire of E3 ligases, multiple isoforms of several core ERAD components, and a number of CDC48-UN complex adaptations, suggesting heightened complexity in photosynthetic eukaryotes (Fig. 5a)^84,86^. Plants also possess an extended set of UBX-type Cdc48 adaptors, termed plant UBX domain-containing proteins (PUX), indicating substantial diversification of Cdc48-dependent processes in plants. These evolutionary adaptations may be related to plants’ sessile lifestyle, enabling better management of diverse environmental challenges.

Genome-wide analysis revealed that the model plant Arabidopsis invests nearly 6% of its proteome (~ 1600 genes) in the UPS, with most components being ubiquitin ligases (> 1400 genes, as noted earlier), enabling extraordinary specificity and complexity of proteolytic regulation in plants^3,87^. Although some of these E3s have obvious yeast and animal counterparts, many are likely plant-specific and thus deliver regulation that is unique to plants^3^. In line with this broader picture, several plant-specific E3 ligases linked to ERAD have been identified^88,89^. For example, the cytosolic ERAD-mediating RING finger protein (EMR), identified in Arabidopsis, affects brassinosteroid hormone signalling under ER stress conditions^90^; while the ER membrane-localised salt tunicamycin-induced RING finger protein (STMIR), identified in *Medicago falcata*, relieves ER stress during salt stress^91^. Both of these E3s work together with the E2 conjugating enzyme UBC32, but mechanistic details remain to be established.

One or more Arabidopsis homologues of the core ERAD components Hrd1, Hrd3, Yos9 and Ubc6 (respectively termed Hrd1a and Hrd1b, Hrd3a [also known as EMS-mutagenized bri1 suppressor 5, EBS5], OS-9 [also known as EBS6], and UBC32) have been identified and studied^86,92–95^. These components play key roles in the degradation of misfolded ER proteins, including two ER-retained mutant forms of the brassinosteroid receptor, brassinosteroid-insensitive 1 (BRI1)^84^. The N-glycan signal that marks misfolded glycoproteins for degradation is similar to that in yeast and animals^96^. Multiple isoforms of Der1 also exist in Arabidopsis (Der1, Der2.1 and Der2.2), though Cue1 and Usa1 homologues are lacking^97^. However, biochemical evidence for Der1 interaction with Hrd1 in plants is missing; this may be related to the lack of Usa1 in plants, which in yeast stabilises the Der1-Hrd1 association. Interestingly, several plant-specific Hrd1 interactions have been identified. The EBS7 protein is highly conserved in plants, is localised in the ER membrane, and helps to maintain stability of Hrd1^98^. This stabilising function of EBS7 is supported by two closely-related proteins associated with Hrd1 (PAWH1 and PAWH2), which are similarly well conserved in plants^94^. In spite of the existence of these plant-specific factors, the core ERAD complex (comprising Hrd1a, Hrd3a/EBS5 and OS-9/EBS6) appears well conserved in plants, as structural prediction reveals a folding pattern that is highly similar to that in yeast (Fig. 5b). However, attempts to incorporate EBS7 and PAWH1 into this structural model yielded low scores.

Recent observations in rice revealed a close connection between signal peptide peptidase (SPP)-like proteins and ERAD^99^. These are aspartyl proteases that mediate intramembrane protein cleavage processes, and the new findings parallel earlier observations made in mammals^23,100^. The rice proteins were linked to thermotolerance in plants, making them promising targets for crop improvement.

Although Cdc48 is highly conserved in plants, it does show a number of plant-specific adaptations. In Arabidopsis, there are five homologues of Cdc48 (A to E, with Cdc48A apparently playing the major role), two homologues of Npl4, and three homologues of Ufd1, again suggesting increased complexity. Recent work revealed that Cdc48A displays distinct domain dynamics and engages Npl4 in a unique manner^101,102^. Unlike in the yeast system, plant Npl4 and Ufd1 do not form an obligate heterodimer; instead, Npl4 can independently associate with Cdc48A to mediate target degradation, although combined action of the adaptors is complementary^101^.

### Mitochondrial protein degradation pathways

Mitochondria are essential energy suppliers in eukaryotic cells^103^. They originated from alphaproteobacteria via an ancient endosymbiotic event, and thus are surrounded by a double membrane system. Their formation and the delivery of their vital metabolic activities require around 1000–1500 nucleus-encoded proteins that are imported post-translationally following synthesis on cytosolic ribosomes, as well as a small number of organelle-encoded proteins. The translocon of the outer mitochondrial membrane (TOM) is a multiprotein complex that forms the entry gate of the protein import pathway^104,105^. Owing to the central role played by mitochondria in cellular respiration and metabolism, protein abnormalities in these organelles can severely hamper cellular functions with dire consequences, and are associated with multiple human pathologies^106,107^. Ubiquitin-dependent protein degradation helps to maintain mitochondrial quality by removing damaged or misfolded proteins, as well as translocationally-stalled precursor proteins at the TOM complex, to prevent their accumulation and potential toxicity^103^. Such proteolysis additionally plays a role in regulating mitochondrial dynamics by controlling the levels of fusion and fission factors^108^. Thus, understanding the intricacies of these systems is crucial not only from a fundamental perspective but also for its potential to deliver therapeutic interventions.

Mitochondria exhibit two Cdc48-dependent pathways that enable the clearance of misfolded or aberrant proteins, as well as a number of other pathways that do not rely on Cdc48^103,107^. The two relevant pathways are mitochondria-associated protein degradation (MAD) and mitochondrial protein translocation-associated degradation (mitoTAD) (Fig. 4b). The former maintains mitochondrial proteostasis by degrading misfolded or damaged proteins, notably those located in the outer mitochondrial membrane (OMM), while the latter clears precursor proteins trapped in the import channel of the TOM translocon^24–26,103,109^.

### Mitochondria-associated protein degradation (MAD)

Early work established MAD as a stress-responsive, Cdc48/p97-dependent pathway mediating the turnover of mitochondrial outer membrane proteins^24,25^. Multiple ubiquitin ligases have been reported to act in MAD with its action being connected especially with mitochondrial dynamics^110–112^. For instance, the OMM-localised dynamin GTPase fuzzy onion 1 (Fzo1), which controls mitochondrial fusion, is ubiquitinated cooperatively by reverses SPT-phenotype protein 5 (Rsp5, a HECT-type E3 ligase) and mitochondrial distribution and morphology protein 30 (Mdm30, an F-box protein of a SCF-type E3 ligase)^113–116^. The ubiquitination of Fzo1 is tightly controlled and can be reversed by the DUBs ubiquitin-specific proteases 2 and 12 (Ubp2, Ubp12)^113,117^. In addition, the mitochondria-associated F-box protein-1 (Mfb1) has been implicated in remodelling Fzo1 to maintain its competent state^118^. Other OMM proteins (including the protein import receptor Tom70, Msp1 and Mdm34) are also targeted by Rsp5, though this may occur independently of Mdm30^119,120^. Other E3 ligases implicated in MAD include the RING domain proteins Ubr1 and sir antagonist 1 (San1)^112^.

In a remarkable parallel with ERAD, Ubx2 acts in the recruitment of the Cdc48-UN complex to the OMM^112,121–123^. While the VCP/Cdc48-associated mitochondrial stress-responsive 1 (Vms1) protein has also been suggested to act in Cdc48 recruitment, recent evidence suggests that it is involved in ribosome quality control^124,125^. Another component implicated in Cdc48 recruitment for the degradation of MAD substrates is Doa1 (also known as Ufd3)^120^. Doa1 has been shown to bind to the Cdc48 C-terminus^126,127^, but how it affects the degradation of mitochondrial proteins is not fully understood. In a further parallel with ERAD, cytosolic Hsp70/Hsp40 chaperones are involved, presumably maintaining mitochondrial substrates in a non-aggregated state suitable for ubiquitination and Cdc48-dependent extraction^112^. Although it is well established that substrates are extracted via Cdc48, underlying mechanisms had remained unclear, including whether substrates are removed directly from the membrane or through a retrotranslocon. However, recent work supports a role for the core channel component of the protein import machinery (i.e., the Tom40 protein) in the MAD retrotranslocation function (Fig. 4b)^128^.

Besides its role in degrading OMM substrates, the MAD system may also act on proteins of the organellar interior^129,130^. For instance, proteins of the intermembrane space are released via the TOM machinery for proteasomal degradation, although whether Cdc48 participates in this pathway is unclear^131^. Moreover, several studies suggested that the UPS may regulate inner membrane or matrix proteins^132,133^. For example, MAD was proposed to regulate energy metabolism in mammalian cells by targeting the inner mitochondrial membrane, and in particular the succinate dehydrogenase subunit A (SDHA) protein^130^. Another study identified matrix proteins as MAD substrates in yeast, with the ubiquitination occurring preferentially under oxidative stress conditions and MAD activity being critical for cellular fitness under such circumstances^129^. A more recent study presented evidence that ubiquitination enzymes are localised in the yeast mitochondrial matrix, with the manipulation of a specific E2 enzyme causing changes in the ubiquitination of mitochondrial proteins^134^. Evidence that target proteins may be exported (or retrotranslocated) from the mitochondrial interior in a Cdc48-dependent fashion has also been reported^128^.

While MAD has been well characterised in yeast, knowledge on equivalent plant systems remains limited. Recently, a novel cytosolic protein identified in Arabidopsis, transmembrane domain-binding protein for tail-anchored outer membrane proteins (TTOP), was hypothesised to cooperate with Cdc48 in the ubiquitin-dependent removal of tail-anchored receptor components of the mitochondrial TOM complex (and of the equivalent translocon complex in chloroplasts; see later), facilitating their delivery to the proteasome^135^. The TTOP protein possesses an N-terminal ubiquitin-like (UBL) domain that may mediate proteasomal docking.

### Mitochondrial protein translocation-associated degradation (mitoTAD)

An active state of the TOM translocon is essential for mitochondrial biogenesis and respiration, but can be lost if the translocation channel is occluded by trapped precursor protein. Such clogging can further induce the accumulation of unimported precursor proteins, resulting in cytosolic proteostatic stress, and is highly toxic for the cell^106^. The mitoTAD pathway provides a continuous surveillance mechanism that clears translocationally-stalled precursors so as to avoid such damaging scenarios^26,109^.

As in the MAD pathway, Rsp5 provides E3 ligase activity, in this case upon anchoring at the Tom70 receptor of the TOM translocon^136^. Whether other E3 ligases are also involved, and the identity of the relevant E2 enzymes, remain open questions. Again, the Ubx2 component previously identified as a key participant in ERAD is involved. A mitochondrial sub-pool of Ubx2 is specifically bound to the TOM complex following insertion into the OMM via the Tom70 receptor^26^. The Ubx2 protein recruits Cdc48 with its Ufd1-Npl4 cofactor complex to the TOM complex, paralleling its role in ERAD pathways. Thus recruited, the Cdc48 motor promotes the proteasomal degradation of arrested precursors by extracting them from the translocon channel (Fig. 4b). Exactly how the problematic precursor proteins are recognised for ubiquitination is unclear, though it may depend on the time a precursor remains at the TOM complex^105,109^. The DUB Ubp16 also associates with the TOM complex, and may counteract the effects of Rsp5 by removing inappropriate ubiquitin marks from slowly importing but normal precursor proteins^136^.

In a parallel, Cdc48-independent pathway, ubiquitinated precursors stalled at the TOM complex are delivered to the proteasome by the Pth2-Dsk2 system^136^. Peptidyl-tRNA hydrolase 2 (Pth2) provides a membrane-embedded docking site at the TOM complex for the recruitment of dominant suppressor of Kar1 2 (Dsk2), a UBA domain protein that mediates substrate transfer towards the proteasome. Moreover, while the mitoTAD pathway appears to operate under normal conditions, during stressful conditions an additional clearance system comes into play. The mitochondrial compromised protein import response (mitoCPR) system also operates to remove translocationally-stalled preproteins from the TOM complex, but it does so in a Cdc48 independent manner with a different AAA+ ATPase, Msp1, acting as the motor instead^110,137^. Abnormalities in mitochondrial protein import induce the expression of citrinin-sensitive knockout protein 1 (Cis1), which connects Msp1 to Tom70 so as to promote the extraction of stalled preproteins.

Interestingly, mitochondria and the ER cooperate to clear mistargeted tail-anchored proteins from the OMM. Msp1 plays a key role in this process by recognising and removing these mislocalized tail-anchored proteins^138,139^. Once identified by Msp1 at the OMM, the substrates are ubiquitinated by the ERAD E3 ligase Doa10 at the ER-mitochondria contact sites. Following ubiquitination, the substrates are extracted from the membrane by Cdc48 and delivered to the proteasome for degradation^138^.

### Chloroplast protein degradation pathways

Chloroplasts are the defining organelles of plants and algae^140^. They are equipped with molecular machines for photosynthesis, the vital process whereby carbon dioxide is fixed into organic molecules with the release of molecular oxygen. Like mitochondria, chloroplasts are of prokaryotic and endosymbiotic origin, though in this case the endosymbiont was a cyanobacterium. Reflecting their similar origins, chloroplasts share several features with mitochondria, including a double bounding membrane and the need for protein import systems in these membranes^141^. The protein import machinery is composed of translocons in the outer and inner chloroplast membranes (TOC and TIC, respectively)^142,143^. Thousands of nucleus-encoded proteins are translocated via the TOC-TIC system in order to build essential molecular systems in different subcompartments of the organelle. Remarkably, the outer envelope membrane (OEM)-localised TOC system is directly regulated by the cytosolic UPS, enabling centralised control over protein import in response to internal and external cues^27,144^. This chloroplast-associated protein degradation (CHLORAD) system was identified in *Arabidopsis thaliana* using a combination of genetics and proteomics, but it is widely conserved in plants.

In vivo analyses revealed that CHLORAD plays essential roles in plant growth and development^141,144^. By exerting control over protein import, the system helps to shape the organellar proteome and functions, which is especially important during developmental transitions such as fruit ripening^27,145^. Notably, the system helps to limit harmful reactive oxygen species (ROS) formation under adverse environmental conditions by controlling photosynthesis, thereby contributing positively to stress tolerance^144^. These observations suggest that CHLORAD has considerable potential as a technology for crop improvement.

### Ubiquitin-dependent degradation of the chloroplast protein import machinery

Key components of the CHLORAD system are suppressor of plastid protein import1 locus 1 (SP1) and SP2, which are both integral OEM proteins (Fig. 4c)^27,146,147^. The former is a RING-type E3 ligase that ubiquitinates TOC proteins. It has two transmembrane spans, an intermembrane space domain which directly interacts with its TOC targets, and a C-terminal RING domain facing the cytosol. Unlike the E3 ligases involved in ERAD (e.g., Hrd1), SP1 lacks the capacity to also form a retrotranslocon. Instead, SP2, which is an outer membrane protein 85 (Omp85)-related β-barrel channel, is believed to fulfil the retrotranslocon requirement for TOC protein extraction^146^. Two homologues of SP1, termed SP1-like 1 (SPL1) and SPL2, help to fine-tune CHLORAD activity, with SPL2 exhibiting partial functional redundancy with SP1, and SPL1 functioning as a negative regulator of SP1^148^. The identity of the E1 and E2 enzymes involved in CHLORAD is presently unknown.

Involvement of Cdc48 in this pathway reflects the fact that TOC components are membrane-anchored proteins that therefore require extraction. Its role was revealed by the interaction of Cdc48 with the established CHLORAD system components and targets, and by elevated TOC accumulation upon Cdc48 inhibition in plants^146^. Multiple Cdc48 adaptor proteins exist in plants, and recent work uncovered an important role for one of these in CHLORAD. The plant UBX domain-containing protein 10 (PUX10) is localised in the chloroplast OEM, where it mediates recruitment of Cdc48 to the chloroplast surface as well as delivery of ubiquitinated substrates to Cdc48 (Fig. 4c)^149^. This presents a striking parallel with the role played by Ubx2 in the ER and mitochondria, and in fact, PUX10 is a homologue of Ubx2. Indeed, the two proteins share notable sequence and topological similarity, with both possessing cytosol-facing UBX and UBA domains that bind to Cdc48 and ubiquitinated substrate proteins, respectively.

### Ubiquitin-dependent degradation of other chloroplast proteins

Interestingly, homologues of the Cdc48 adaptor proteins Ufd1 and Npl4 also exist in Arabidopsis, although whether they are similarly involved in CHLORAD-dependent degradation of the import machinery remains to be determined. However, the stabilisation of chloroplast proteins (including components of the photosynthetic apparatus, in the stroma and thylakoids) was unexpectedly observed in mutants for these adaptor proteins, pointing to a broader role for Cdc48 and the UPS in chloroplast protein degradation^150^. This conclusion was supported by the independent detection of ubiquitinated proteins inside chloroplasts using proteomic and immunological approaches, and by the impaired degradation of photosynthetic proteins in SP2- and Cdc48-deficient plants^151^. While some of these discoveries have proved controversial^152,153^, they do raise important and intriguing questions about how ubiquitination connects with organellar proteostasis, related to the site of ubiquitination, the identity and localisation of relevant ubiquitination machinery, and the mechanisms that may enable delivery of ubiquitinated organellar targets to the cytosol for proteasomal degradation. As with the corresponding mitochondrial results discussed earlier, further investigations are necessary to clarify the significance of these observations and to uncover the underlying mechanisms^153^.

### Comparison of the different systems

The different organelle-specific proteolytic pathways highlighted above share many similarities, not least of which is the fact that they all incorporate ubiquitination enzyme cascades and are based around the Cdc48 retrotranslocation motor for the delivery of target proteins to the 26S proteasome. However, there are also several areas where the pathways show notable differences, for example, in relation to the identity and properties of the respective substrates, E3 ligases, retrotranslocons, and Cdc48 adaptor proteins or recruitment factors.

### An evolutionary perspective on Cdc48-dependent proteolytic pathways

Archaea, including the Asgard archaea which are the closest prokaryotic relatives of eukaryotes, possess both Cdc48 and the 20S proteolytic particle, as well as a primitive precursor of the eukaryotic ubiquitination cascade^154–156^. Moreover, it has been shown that some archaea possess a proteolytic system comprising the 20S core particle capped with Cdc48, with the latter believed to act analogously to the AAA+ subunits of the 19S particle in eukaryotic proteasomes^156,157^. Thus, one can infer that during eukaryogenesis – in line with the elaboration of the ubiquitination system and concomitant appearance of the 19S particle, as well as increasing cellular complexity linked to formation of the endomembrane system and organelles – Cdc48 was repurposed to fulfil upstream functions preceding proteasomal action, including target protein extraction from membranes.

The emergence of complex internal compartmentalisation in eukaryotes presented new challenges for protein homeostasis which Cdc48 was well suited to address, with its increasingly diverse functions being supported by a plethora of different adaptor proteins. It appears likely that ERAD evolved in conjunction with the ER itself, owing to the organelle’s central role in the secretory pathway. While the timing of mitochondrial evolution has been debated, recent evidence supports this occurring relatively late (after the appearance of the endomembrane system)^158^, suggesting that MAD may have adapted some of the systems and components (such as Cdc48 and Ubx2) already established in ERAD^112,122^.

The later acquisition of a second highly-proteinaceous endosymbiotic organelle in photosynthetic eukaryotes, the chloroplast, imposed additional demands on cellular proteostasis. This necessitated the evolution of another dedicated UPS pathway, CHLORAD, to regulate the new organelle, and again this appears to have co-opted components from other pathways (notably, Cdc48 and PUX10/Ubx2)^27,146,149^. However, the resulting co-existence of multiple CDC48-dependent pathways introduced unique challenges for plants related to substrate specificity and regulatory coordination, adding further complexity to cellular proteostasis networks. To meet these demands, two evolutionary adaptations likely occurred: (i) pre-existing systems, including ERAD and MAD, may have undergone functional specialisation or diversification in plants, to enhance target selection specificity and distinguish the different organelles (e.g., see earlier discussion of ERAD in plants); (ii) the CHLORAD pathway developed bespoke mechanisms that are tailored to meet the unique requirements of chloroplasts.

### The E3 ligase and channel functions are delivered in different ways

Given that E3 ligases confer substrate specificity, it is not surprising that these components constitute a primary area of differentiation between the three focal pathways. Each system has its own unique set of E3 ligases, enabling it to act selectively on a defined set of targets in the relevant organelle, or organelle subcompartment, and under appropriate conditions. A remarkable feature of the ERAD E3 ligases, Hrd1 and Doa10, is their multifunctionality: because their membrane-embedded segments are polytopic in character (comprising eight and 14 transmembrane segments, respectively), they additionally have the capacity to contribute to retrotranslocon formation or substrate engagement within the membrane^46,48,70,71^. In contrast, the E3s so far identified in MAD, mitoTAD and CHLORAD (e.g., Rsp5, Mdm30, and SP1) tend to have simpler topology or are cytosolic, and so lack the capacity to form deep membrane cavities for substrate export or engagement^27,114,115^. In the case of CHLORAD, this situation likely led to the recruitment of SP2 as a separate, dedicated channel component^146^. The SP2 protein is a 16-stranded β-barrel protein of the Omp85 superfamily, and as such is related to various other protein translocation factors in bacteria^159^. Recruitment of a factor with such obvious prokaryotic origins to a system that is ostensibly eukaryotic in character is quite remarkable. Contrastingly, the channel-forming components involved in ERAD are formed of α-helical transmembrane segments.

In the ER and chloroplasts, protein import (for new protein biogenesis) and protein export (for proteolysis via ERAD and CHLORAD) occur via separate pathways: protein import employs the Sec61 and Toc75 translocons, respectively^141,160^, whereas protein export is mediated via the Hrd1 and SP2 components as discussed above. In mitochondria, the situation is rather different, as the MAD system appears to utilise the same channel protein for both import and export functions^128^. The relevant component is Tom40, which is well known as the core channel-forming component of the TOM complex^104,105^. Whether this reflects a fundamental difference between MAD and the other systems, or particular characteristics of the Tom40 protein that enable it to function more flexibly, is unclear. Regardless, it is interesting to note that Tom40 is structurally similar to SP2 in that it is also a β-barrel protein, albeit not a member of the Omp85 superfamily like SP2. This suggests that β-barrel proteins may be well-suited to the retrotranslocation function in these organelles. Even though the import and export channel components of CHLORAD are different proteins (i.e., Toc75 and SP2), it is worth noting that they are nonetheless related since they both belong to the Omp85 superfamily^146^.

### Cdc48 recruitment to the organelle surface involves similar factors

The different systems described in this article rely on the pulling force exerted by Cdc48 to extract target proteins prior to their degradation by the 26S proteasome. Notably, the way in which Cdc48 is recruited to the surface of the respective organelle presents some interesting commonalities and differences. The Ubx2 protein acts in Cdc48 recruitment in ERAD, MAD and mitoTAD^26,75,76,109,122^. That said, Doa1 is also reported to contribute to this function in MAD, suggesting that Cdc48 recruitment in this system has additional requirements or control systems^112,120^. While the PUX10 protein fulfils the analogous role in the CHLORAD pathway, it should be noted that PUX10 is closely related to Ubx2 (with cytosolically oriented UBX and UBA domains) and presumably functions in a very similar way^149^.

With regard to substrate recognition and processing at Cdc48, the Ufd1 and Npl4 adaptor proteins appear to participate in most of the discussed systems, though have undergone notable diversification in plants^26,79,101,112,150^. Although these adaptors have not yet been linked to the regulation of chloroplast protein import, corresponding mutants have been shown to affect the abundance of internally-localised chloroplast proteins^150^.

### The systems face different topological challenges

Another significant area of differentiation relates to the fact that the systems face distinct topological challenges, owing to the fact that the ER is formed of just a single membrane, whereas the endosymbiotically-derived organelles are both surrounded by two bounding membranes. In spite of the ER’s relative simplicity in this regard, ERAD is nonetheless remarkably complex with dedicated pathways operating at both sides of, and within, the membrane (i.e., ERAD-L, -C and -M)^23^. In the former, the target proteins are dislocated to the ER surface prior to ubiquitination. While MAD and CHLORAD have thus far been shown to target mainly proteins of the outer membrane, there is accumulating evidence that these or related systems may additionally process proteins located internally, in the intermembrane space, inner membrane, or beyond, although the underlying molecular mechanisms are poorly understood^129,130,150,151^. In fact, the initial events governing target selection and delivery towards the retrotranslocon are poorly characterised in all cases except for ERAD-L. The latter system utilises internal proteins including Yos9, Hrd3 and Mnl1-Pdi1 to ensure proper substrate recruitment and processing prior to export^23,49,51,52,55^.

### Connections to organelle-specific protein import pathways are prevalent

Lastly, it is noteworthy that all of the systems discussed here display strong connections to the main protein translocation or import system of the relevant organelle. The ER and the mitochondrion both employ Cdc48-based systems to clear arrested precursor proteins from the translocon channel (Sec61 and Tom40, respectively), thereby preventing permanent damage to the protein translocation apparatus as well as broader proteostasis-related cytotoxicity – these are the ERAD-T and mitoTAD systems, respectively^26,37^. The post-translational nature of mitochondrial protein import may make translocon clogging an especially acute problem for this organelle. Moreover, subunits of the ER and mitochondrial translocons are themselves targeted by ERAD and MAD under certain circumstances^22,120^.

At present, there is no evidence that arrested precursors are similarly removed from the TOC translocon in chloroplasts, which may be related to the fact the chloroplast protein import machinery is much less prone to clogging (due perhaps to greater channel flexibility or a stronger import motor)^161,162^. Nonetheless, given the presence of apparently suitable apparatus adjacent to the translocon (i.e., Cdc48 and PUX10), it would not be so surprising if analogous translocon-clearing mechanisms were found to exist in chloroplasts. Regardless, the primary role of CHLORAD appears to be direct regulation of the TOC machinery, enabling its dynamic reconfiguration in response to internal and environmental signals^27,140,141^. In this way, CHLORAD exerts control over the proteome of the organelles and contributes to their remarkable morphological, functional and developmental plasticity.

### Implications in human health and agriculture

Our understanding of the importance of ERAD in human health and disease continues to advance^33,34^. Deletion of highly-conserved ERAD components such as Hrd1 and SEL1L (Hrd3) results in embryonic lethality in mice, and mutations in these components are linked to protein-misfolding disorders, metabolic diseases (e.g., diabetes), and several neurodegenerative pathologies, including amyotrophic lateral sclerosis and Parkinson’s disease in humans^34,163,164^. Reduced Hrd1 levels can result in the accumulation of misfolded proteins and have been linked to Alzheimer’s disease in humans^165^. By removing damaged, misfolded or aggregated proteins, the core elements of ERAD play pivotal roles in cellular health and present significant therapeutic potential^34^. Though research efforts have rightly focused on homologous mammalian systems, we suggest that also studying the expanded network of ERAD components in plants, for example via comparative structural and interface analyses of plant and human Hrd1 complexes, may offer further valuable insights or opportunities for therapeutic intervention. Plant-derived perspectives for developing novel therapies against ERAD-related human diseases could include identifying additional regulatory layers governing Hrd1 stability, potentially via modification of SEL1L-Hrd1 binding.

Manipulation of plant ERAD components further offers possibilities to improve crop performance under biotic and abiotic stress conditions^95,166,167^. ERAD-related E2 and E3 enzymes identified in rice and wheat have recently been shown to modulate the brassinosteroid signalling pathway, a key hormonal system that promotes cell expansion and organ growth^88,168^. Targeted manipulation of these E2/E3 enzymes influenced grain size and weight, making ERAD a promising target for crop improvement^88,168^.

Research in the model plant Arabidopsis uncovered a vital role for CHLORAD in reconfiguring the chloroplast protein import machinery, which in turn facilitates the organelle-proteome dynamics responsible for the striking structural and functional changes to the organelles (i.e., chloroplasts and other plastid types) that underpin major plant development transitions^27,146^. This knowledge led to the realisation that CHLORAD might be manipulated to beneficially alter crop growth in different ways. Proof of principle in this regard was provided in tomato (where fruit ripening is characterised by dramatic transformation of chloroplasts into bright-red chromoplasts enriched in carotenoid pigments): plants with reduced CHLORAD activity showed delayed fruit ripening, whereas those with increased activity showed accelerated ripening^145^. Fruit ripening is trait of major agronomic importance, and such manipulation could, for example, be implemented to promote shelf life and reduce waste. Depending on the developmental context, CHLORAD manipulation might similarly be altered to modify starch accumulation in seeds or to prolong photosynthetic activity in the leaves to improve yields. As noted earlier, CHLORAD also has important functions in stress tolerance (involving limitation of excessive photosystem activity and consequent ROS production)^144^, further extending the potential applications of CHLORAD as a technology for crop improvement.

### Concluding remarks

Targeted protein degradation by the UPS is essential for the maintenance of a balanced and responsive proteome and for the prevention of toxic protein accumulation, and thus has a central role in ensuring a huge diversity of cellular functions^1–3,169^. Even though the main components of the UPS are restricted to nucleocytosolic compartments, the Cdc48-dependent pathways described herein bring numerous proteins localised in diverse, membrane-bound subcompartments within reach, vastly extending the system’s scope. As discussed, although each pathway is based around some common principles and processes (i.e., ubiquitination cascades, ATP-powered retrotranslocation, and proteasomal degradation in the cytosol), they are nonetheless exquisitely tailored to meet local, organelle-specific demands. Notable differences between the systems pertain to their substrates, E3 ligases, retrotranslocons, and Cdc48 adaptor or recruitment proteins; to the combination of different functions within multifunctional proteins in the case of the ER; and to the utilisation of components of clear prokaryotic origin in the case of the endosymbiotic organelles. A fascinating and recurring theme with all of the systems discussed here is a strong functional connection with the respective organelle’s protein import machinery, underscoring how closely integrated different proteostatic processes are. Despite the remarkable advances made recently in our understanding of ubiquitin-dependent proteolysis at organelles, many unanswered questions remain (Box 3), and so the field promises to be an exciting area of discovery for the foreseeable future.

Box 3 Future challenges Salient questions concern the molecular details of target selection, ubiquitination and presentation to Cdc48, and the regulatory steps governing these processes (e.g., via deubiquitination and cofactors). The role of ubiquitin chain topology warrants more careful attention, as does the interplay with other processes to deliver balanced proteostasis at a cellular level.

Another challenging area that requires further investigation is the crosstalk among the different Cdc48-dependent pathways within the cell, especially in plants where the additional CHLORAD pathway presents heightened challenges regarding substrate selectivity, coordination and regulatory governance. Evolutionary questions concerning the origins of the different pathways, especially those in the endosymbiotic organelles, are fascinating and also not well explored.

Emerging evidence of intra-organellar ubiquitination in mitochondria and chloroplasts challenges long-standing paradigms related to proteolytic control in endosymbiotic organelles. If taken at face value, these data raise additional intriguing questions related to the identity and localisation of the relevant ubiquitination machinery, how such processes might be regulated, and how the targets, once modified, are exported across the double bounding membrane to enable proteasomal degradation in the cytosol.

In the future, improved understanding of these crucial proteolytic systems may provide new avenues for treating medical conditions linked to protein misfolding and aggregation, such as neurodegenerative disorders and aging, or lead to innovative approaches to improve crop yield, stress resistance, and plant health, which will be crucial for addressing food security challenges in the face of climate change.
